# Clinical features and prognostic factors in patients with microvascular infiltration of hepatocellular carcinoma: Development and validation of a nomogram and risk stratification based on the SEER database

**DOI:** 10.3389/fonc.2022.987603

**Published:** 2022-09-14

**Authors:** Dashuai Yang, Mingqiang Zhu, Xiangyun Xiong, Yang Su, Fangrui Zhao, Yong Hu, Guo Zhang, Junpeng Pei, Youming Ding

**Affiliations:** ^1^ Department of Hepatobiliary Surgery, Renmin Hospital of Wuhan University, Wuhan, China; ^2^ Department of Gastrointestinal Surgery, Tongji Hospital, Tongji Medical College in Huazhong University of Science and Technology, Wuhan, China; ^3^ Department of Oncology, Renmin Hospital of Wuhan University, Wuhan, China; ^4^ Department of Orthopedics, Renmin Hospital of Wuhan University, Wuhan, China; ^5^ Department of neurosurgery, Renmin Hospital of Wuhan University, Wuhan, China

**Keywords:** hepatocellular carcinoma, microvascular invasion, cancer-specific survival, nomogram, risk stratification, prognostic model

## Abstract

**Background:**

The goal is to establish and validate an innovative prognostic risk stratification and nomogram in patients of hepatocellular carcinoma (HCC) with microvascular invasion (MVI) for predicting the cancer-specific survival (CSS).

**Methods:**

1487 qualified patients were selected from the Surveillance, Epidemiology and End Results (SEER) database and randomly assigned to the training cohort and validation cohort in a ratio of 7:3. Concordance index (C-index), area under curve (AUC) and calibration plots were adopted to evaluate the discrimination and calibration of the nomogram. Decision curve analysis (DCA) was used to quantify the net benefit of the nomogram at different threshold probabilities and compare it to the American Joint Committee on Cancer (AJCC) tumor staging system. C-index, net reclassification index (NRI) and integrated discrimination improvement (IDI) were applied to evaluate the improvement of the new model over the AJCC tumor staging system. The new risk stratifications based on the nomogram and the AJCC tumor staging system were compared.

**Results:**

Eight prognostic factors were used to construct the nomogram for HCC patients with MVI. The C-index for the training and validation cohorts was 0.785 and 0.776 respectively. The AUC values were higher than 0.7 both in the training cohort and validation cohort. The calibration plots showed good consistency between the actual observation and the nomogram prediction. The IDI values of 1-, 3-, 5-year CSS in the training cohort were 0.17, 0.16, 0.15, and in the validation cohort were 0.17, 0.17, 0.17 (*P*<0.05). The NRI values of the training cohort were 0.75 at 1-year, 0.68 at 3-year and 0.67 at 5-year. The DCA curves indicated that the new model more accurately predicted 1-year, 3-year, and 5-year CSS in both training and validation cohort, because it added more net benefit than the AJCC staging system. Furthermore, the risk stratification system showed the CSS in different groups had a good regional division.

**Conclusions:**

A comprehensive risk stratification system and nomogram were established to forecast CSS for patients of HCC with MVI.

## Introduction

Hepatocellular carcinoma (HCC) is the main histological type (approximately 90%) of primary hepatocellular carcinoma (1). HCC rank as the sixth most frequent malignancy and the third leading cause for cancer-related mortality worldwide (2).The 5-year survival rate for HCC is only 18% (3).

Currently, surgery is the preferred treatment for HCC, but the high postoperative recurrence and low long-term survival remain to be solved (4, 5). Most HCC patients are losing surgical treatment due to the advanced stage at the time of diagnosis. Different subtypes and molecular heterogeneities of HCC make their clinical prognosis significantly different (6). Microvascular infiltration (MVI) is mainly a nest of cancer cells seen microscopically in the lumen of the blood vessels lining the endothelium, mainly in the branches of the portal vein next to the cancer (7). Most patients with early-stage relapsed liver cancer eventually pathologically MVI positive, which is one of the key prognostic factors for recurrence after liver resection or liver transplantation in HCC patients (8–12). Further studies of pathological grading of MVI found that the higher the grouping level of MVI, the shorter the median survival of HCC patients (13, 14). At the same time, tumor morphology, diameter, and number are important factors in predicting vascular infiltration (15–17). Therefore, HCC patients with MVI require personalized predictive models. Research teams have established clinical predictive models that combine clinical features, laboratory indicators, and imaging features to more accurately predict MVI (18–22).

It should be noted that the nomogram has been extensively used to predict the prognosis of cancer patients, driving personalized medicine and helping clinicians to predict prognosis (23–25). There have been many studies on prediction models for liver cancer, but few studies have been done to construct a nomogram to predict the prognosis of HCC patients with microvascular invasion. This study aimed to establish prediction models for CSS in HCC patients with MVI and to verify their predictive performance.

## Patients and methods

### Patients selection and study variables

SEER*Stat 8.3.6 software was applied to extract data on patients diagnosed with hepatocellular carcinoma and microvascular infiltration from the SEER18 registry database (1975-2019) from 2010-2015 and clinically relevant data. This study used the National Cancer Institute Surveillance, Epidemiology and End Results database, which contains patient follow-up information and is updated once a year. Inclusion criteria: (a) primary hepatocellular carcinoma; (b) complete follow-up information; (c) clear cause of death. Exclusion criteria: (a) unknown tumor primary location; (b) lack of follow-up information; (c) unknown tumor infiltration; (d) unknown cause of death or death from other causes; (e) unknown tumor stage; (f) Distant metastases. The scrubbing process is shown in the flowchart.

### Arrangement of patient data

The study cohort listed the clinical characteristics of hepatocellular carcinoma with microvascular infiltration and the survival characteristics of patients after diagnosis. All variables were expressed as sum of cases and percentages after inclusion of baseline characteristics. Included categories were: age, sex, race, tumor size, tumor number, pathological grade, AJCC stage, AFP value, surgery, chemotherapy, radiotherapy, cause of death classification, month of survival, and survival status. Unfortunately, the database is limited and does not contain information about hepatocirrhosis, lymph node enlargement, pseudocapsule, portal vein tumor thrombosis, HBsAg or HCVab status, ALT, AST, and GGT. Consequently, this study did not include these indicators in the study. In addition, the 7th edition AJCC-TNM staging criteria were adopted.

### Construction and validation of the nomogram model

The Total cases were randomly divided into two groups in the ratio of 7:3, including a training cohort (70% of the total cases) and a validation cohort (30% of the total cases). The training cohort was employed for model construction and the validation cohort for validation. The variables of the establishment of the nomogram were screened by univariate and multivariate Cox regression analyses (*P*<0.05). Consistency index (C-index), subject operating characteristic curve (ROC), calibration curve and decision curve analysis (DCA) were used to validate the nomogram. The C-index was used to reflect the performance and predictive accuracy of the nomogram, while ROC represented the sensitivity and specificity of the nomogram. Calibration plots (1000 self-help weight samples) were plotted for 1, 3 and 5 years to evaluate the predictive power of the model. DCA was plotted to evaluate the clinical utility of the nomogram. In addition, NRI, C-index, IDI were used to evaluate the advantages of the new model.

### A new risk stratification related to the nomogram

Based on the scoring of independent prognostic variables, this study calculated the total score for each patient. The optimal cut-off values were calculated based on the X-tile software to classify patients into low-, middle-, and high-risk groups. The ability of the traditional AJCC staging system and the new risk stratification system to identify different risk groups was compared by KM survival curves.

### Statistical analysis

The data is extracted from the SEER*Stat software (version 8.3.9.2). R version 3.6.3 and related software packages was applied to data analysis. The best cut-off value for the total score was applied by X-Tile (version 3.6.1). Chi-square test was used to assess the differences of distribution of the two cohorts. When *P*-value is less than 0.05, it is statistically significant.

## Results

### Patient characteristics

**Figure 1:**
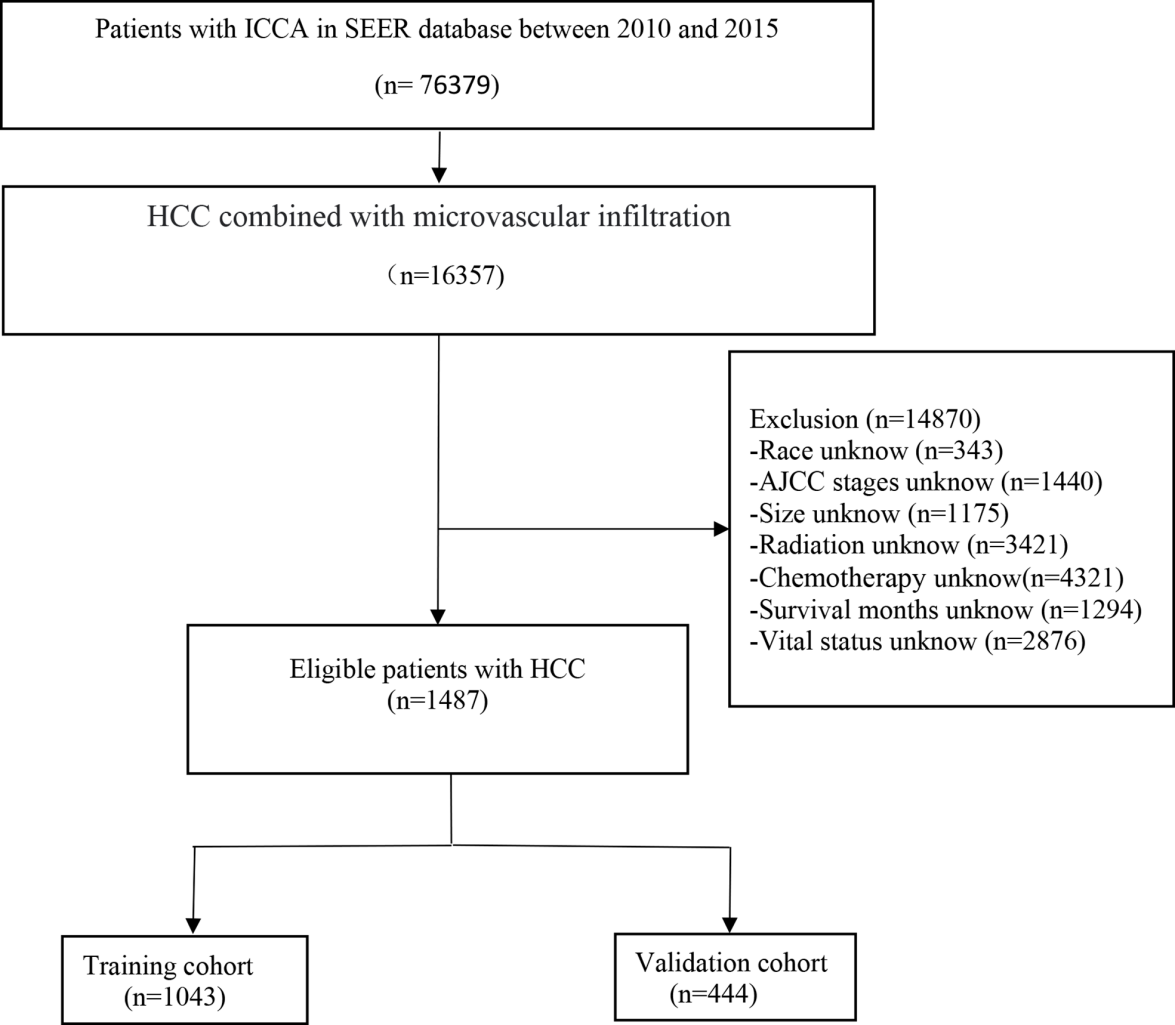
The flowchart of the hepatocellular carcinoma with microvascular infiltration identified in the SEER database.

1487 eligible HCC patients with MVI were included in the research (the training cohort: 1043, the validation cohort: 444) (**Figure 1**). 790 were male and 1297 were white. Nearly half 871 had tumors smaller than 5 cm. most 915 cases were AFP positive, 98 received radiotherapy, and 683 received chemotherapy. **Table 1** summarizes the baseline demographic characteristics and features of patients with hepatocellular carcinoma combined with microvascular infiltration in the training and validation cohorts, with no differences in distribution between the two groups.

**Table 1:** Demographics and clinical and pathology characteristics of the patients with hepatocellular carcinoma and microvascular infiltration.

Variable	Whole population		Training cohort		Validation cohort		P value
	n	%	n	%	n	%	
			1043		444		
Age							
<65	928	62.41%	653	62.61%	275	61.94%	0.80
>65	559	37.59%	390	37.39%	169	38.06%	
Race							
Black	173	11.63%	124	11.89%	49	11.04%	0.44
White	1297	87.22%	908	87.06%	389	87.61%	
Other	17	1.14%	11	1.05%	6	1.35%	
Sex							
F	697	46.87%	489	46.88%	208	46.85%	0.95
M	790	53.13%	554	53.12%	236	53.15%	
AJCC Stages ^a^							
II;	1320	88.77%	925	88.69%	395	88.96%	0.87
III	167	11.23%	118	11.31%	49	11.04%	
Tumor size cm							
0-5	871	58.57%	606	58.10%	265	59.68%	0.57
>5	616	41.43%	437	41.90%	179	40.32%	
AFP							
Positive	915	61.53%	634	60.79%	281	63.29%	0.36
Negative	572	38.47%	409	39.21%	163	36.71%	
Grade^b^							
Well	493	33.15%	358	34.32%	135	30.41%	0.09
Bad	190	12.78%	140	13.42%	50	11.26%	
Unknow	804	54.07%	545	52.25%	259	58.33%	
Number							
1	1198	80.56%	823	78.91%	369	83.10%	0.17
>1	289	19.44%	220	21.09%	75	16.90%	
Surgery						0.00%	
No	822	55.28%	568	54.46%	254	57.21%	0.32
Yes	665	44.72%	475	45.54%	190	42.79%	
Radiation							
Yes	98	6.59%	62	5.94%	36	8.11%	0.13
No	1389	93.41%	981	94.06%	408	91.89%	
Chemotherapy							
Yes	683	45.93%	453	43.43%	214	48.20%	0.10
No	804	54.07%	590	56.57%	230	51.80%	

^a^ AJCC (TNM) Stages, The seventh edition AJCC (TNM) staging system; ^b^ Well, Grade I and II, Bad, Grade III and IV.

### Cox regression to screen independent prognostic factors

Age, AJCC stages, pathological grade, tumor size, number, AFP, surgery, radiation and chemotherapy were significantly identified in univariate COX regression analysis (*P*<0.05). The multivariate analysis showed that tumor size, age, pathological grade, AFP, radiation, AJCC stages, chemotherapy and surgery (*P*<0.05) were independent prognostic factors for CSS which were included in the nomogram (**Table 2**).

**Table 2:** Univariate and Multivariate Cox regression analyses of cancer-specific survival.

Variable	Univariate		*P* value	Multivariate		*P* value
	HR	95% CI		HR	95% CI	
Age						
<65	1.00			1.00		
>65	1.23	1.06-1.43	< 0.05	1.20	1.05-1.36	<0.001
Race						
White	1.00			1.00		
Black	1.17	0.96-1.44	0.14	1.01	0.86-1.25	0.41
Other	1.49	0.77-2.88	0.23	0.86	0.50-1.40	0.23
Sex						
F	1.00			1.00		
M	1.05	0.82-1.01	0.51	1.01	0.89-1.14	0.85
AJCC Stages^a^						
II;	1.00			1.00		
III	1.95	1.59-2.48	< 0.05	1.29	1.06-1.57	< 0.001
Tumor size cm						
0-5	1.00			1.00		
>5	1.80	1.58-2.11	< 0.05	1.87	1.62-2.16	< 0.001
AFP						
Positive	1.00			1.00		
Negative	0.68	0.58-0.79	< 0.05	0.72	0.63-0.82	< 0.001
Grade^b^						
Well	1.00			1.00		
Bad	1.37	1.08-1.77	< 0.05	1.48	1.20-1.81	< 0.001
Unknow	1.67	1.42-1.97	< 0.05	1.15	0.96-1.38	0.12
Number						
1	1.00			1.00		
>1	0.84	0.71-1.01	< 0.05	0.87	0.75-1.02	0.1
Surgery						
No	1.00			1.00		
Yes	0.44	0.38-0.51	< 0.05	0.26	0.21-0.32	< 0.001
Radiation						
Yes	1.00			1.00		
No	0.65	0.49-0.69	< 0.05	1.50	1.81-1.92	< 0.001
Chemotherapy						
Yes	1.00			1.00		
No	1.02	0.88-1.18	0.72	1.90	1.68-2.25	< 0.001

^a^ C (TNM) Stages, The seventh edition AJCC (TNM) staging system; ^b^ Well, Grade I and II, Bad, Grade III and IV.

### Construction and validation of the nomogram

**Figure 2:**
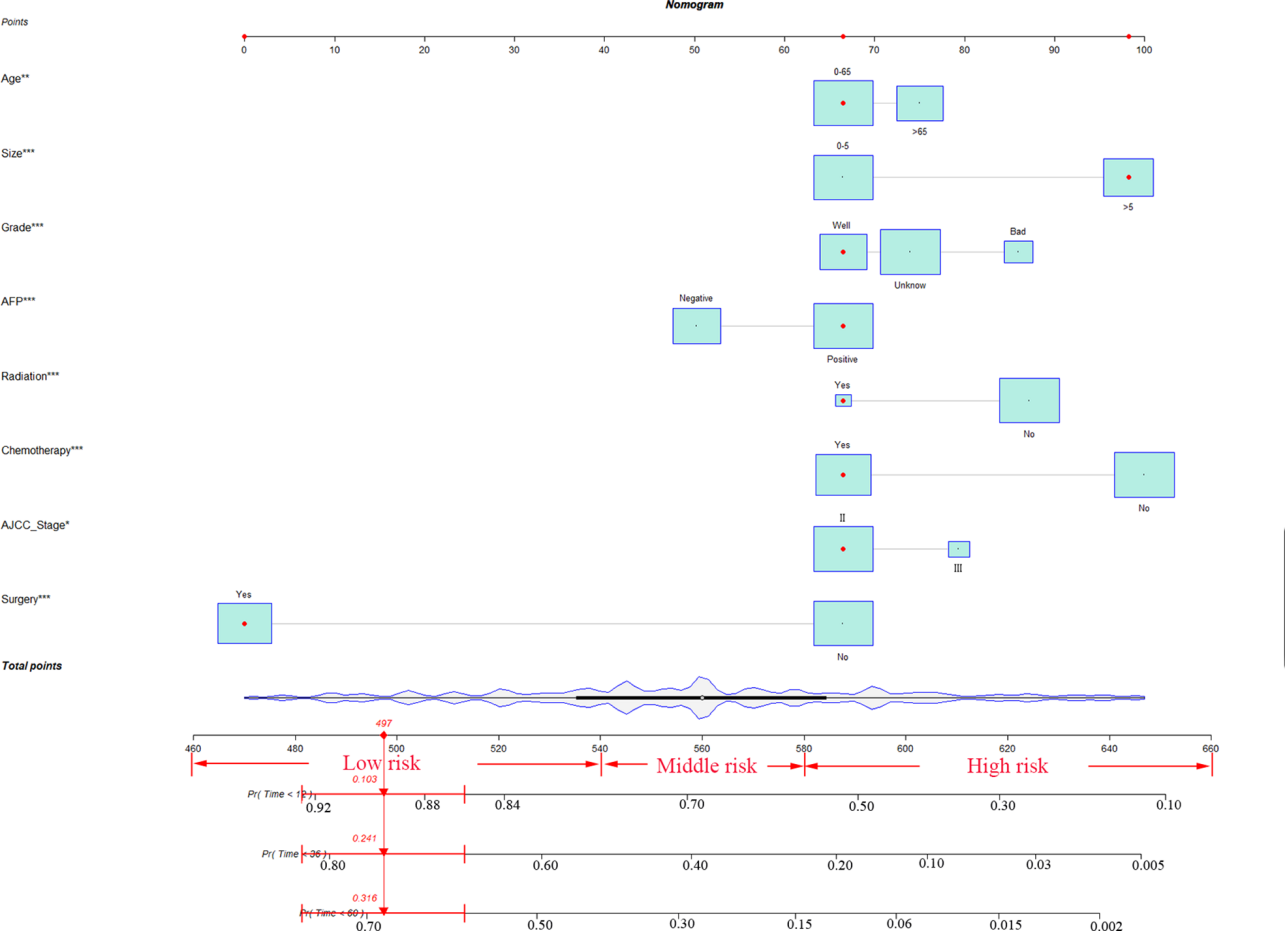
The nomogram for HCC patients with MVI. * P < 0.05, ** P < 0.01, *** P < 0.001, HCC, hepatocellular carcinoma; MVI, microvascular invasion.

**Figure 3:**
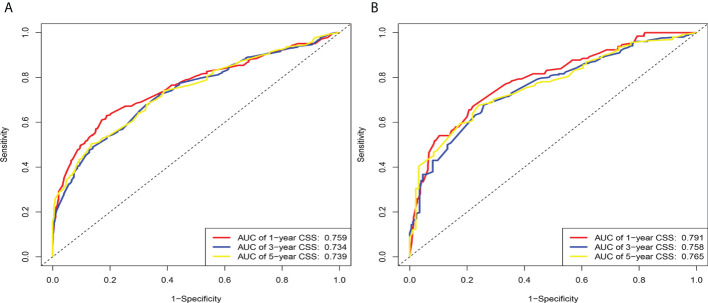
The area under the time-dependent receiver operating characteristic (ROC) curve (td-AUC) based on the nomogram. **(A)** Based on the training cohorts; **(B)** Based on the validation cohorts.

**Figure 4:**
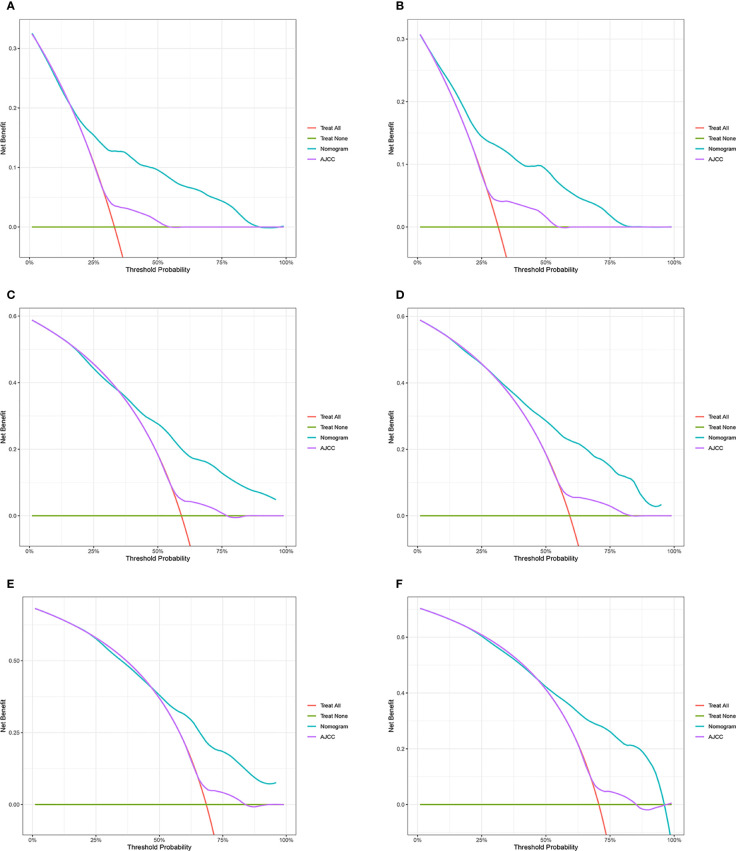
Decision curve analysis of the nomogram and AJCC tumor staging for the cancer-specific survival prediction of HCC patients with MVI. **(A, C, E)** 1-, 3- and 5-year cancer-specific survival benefits based on the training cohorts. **(B, D, F)** 1-, 3- and 5-year cancer-specific survival benefits based on the validation cohort. HCC, hepatocellular carcinoma; MVI, microvascular invasion.

**Figure 5:**
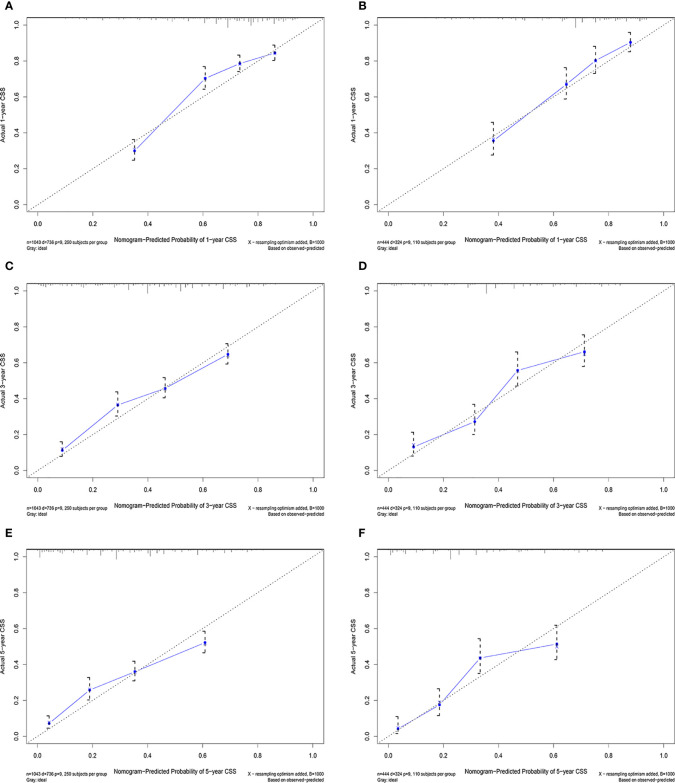
Calibration curves in the training **(A, C, E)** and validation **(B, C, F)** cohorts for 1-year, 3-year, and 5-year cancer-specific survival.

According to the above research results, the nomogram was established to predict CSS at 1, 3, and 5 years for HCC patients with MVI and was validated internally (**Figure 2**). The C-index for the training and validation cohorts was 0.785 (95% CI: 0.741-0.792) and 0.776 (95% CI: 0.716-0.788), respectively. The ROC, and DCA and calibration curves are shown in **Figures 3**–**5**. The ROC curve showed that the 1-, 3-, and 5-year AUC values in the training cohort were 0.759, 0.734, and 0.739, respectively. The 1-, 3-, 5-year td-AUC values in the training cohort were 0.759, 0.734, and 0.739. The td-AUC values of the validation cohort were 0.791 at 1-year, 0.759 at 3-year and 0.765 at 5-year. Furthermore, the DCA curves show good clinical application potential and better positive net benefit in the training and validation cohorts. The calibration curves agreed with the predicted CSS rates at 1, 3, and 5 years.

### Clinical value of the new nomogram compared to the tumor stage based on AJCC staging

**Figure 6:**
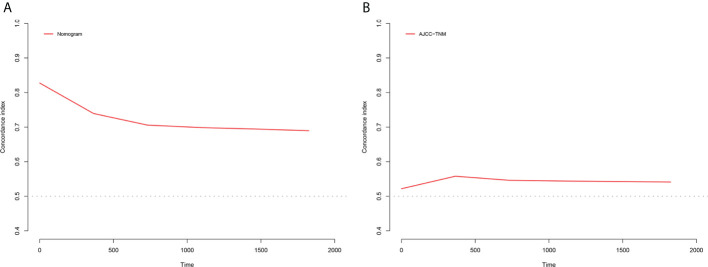
C-index results, **(A)** The C-index based on the nomogram; **(B)** The C-index based on the AJCC tumor staging.

The NRI, IDI and C-index were included in the analysis to compare the advantages and limitations of the nomogram and the traditional AJCC tumor staging system. The results showed that the C-index related to the nomogram was higher than that of the AJCC staging system (**Figure 6**). The IDI values of 1-, 3-, 5-year CSS in the training cohort were 0.17, 0.16, 0.15, and in the validation cohort were 0.17, 0.17, 0.17 (*P*<0.05), indicated that the established nomogram significantly outperformed AJCC staging system. The NRI values of the training cohort were 0.75 (95% CI= 0.59-0.84) at 1-year, 0.68 (95% CI= 0.56-0.83) at 3-year and 0.67 (95% CI= 0.67) at 5-year (**Table 3**). The net benefit of the nomogram was compared to that of the AJCC staging system. The DCA curves showed that the nomogram better predicted 1-, 3-, and 5-year CSS in the all cohorts because the new model showed more net benefit than AJCC staging system.

**Table 3:** NRI and IDI to evaluate the predictive power of the model.

Index	Training cohort		*P* value	Validation cohort		*P* value
	Estimate	95%CI		Estimate	95%CI	
NRI						
1-year CSS	0.75	0.59-0.84		0.66	0.51-0.88	
3-year CSS	0.68	0.56-0.83		0.71	0.53-0.90	
5-year CSS	0.67	0.54-0.78		0.70	0.54-0.95	
IDI						
1-year CSS	0.17	0.13-0.21	< 0.01	0.17	0.12-0.24	< 0.01
3-year CSS	0.16	0.13-0.20	< 0.01	0.17	0.12-0.23	< 0.01
5-year CSS	0.15	0.12-0.19	< 0.01	0.17	0.12-0.25	< 0.01

### Establishment of a stratified risk system based on the nomogram

**Figure 7:**
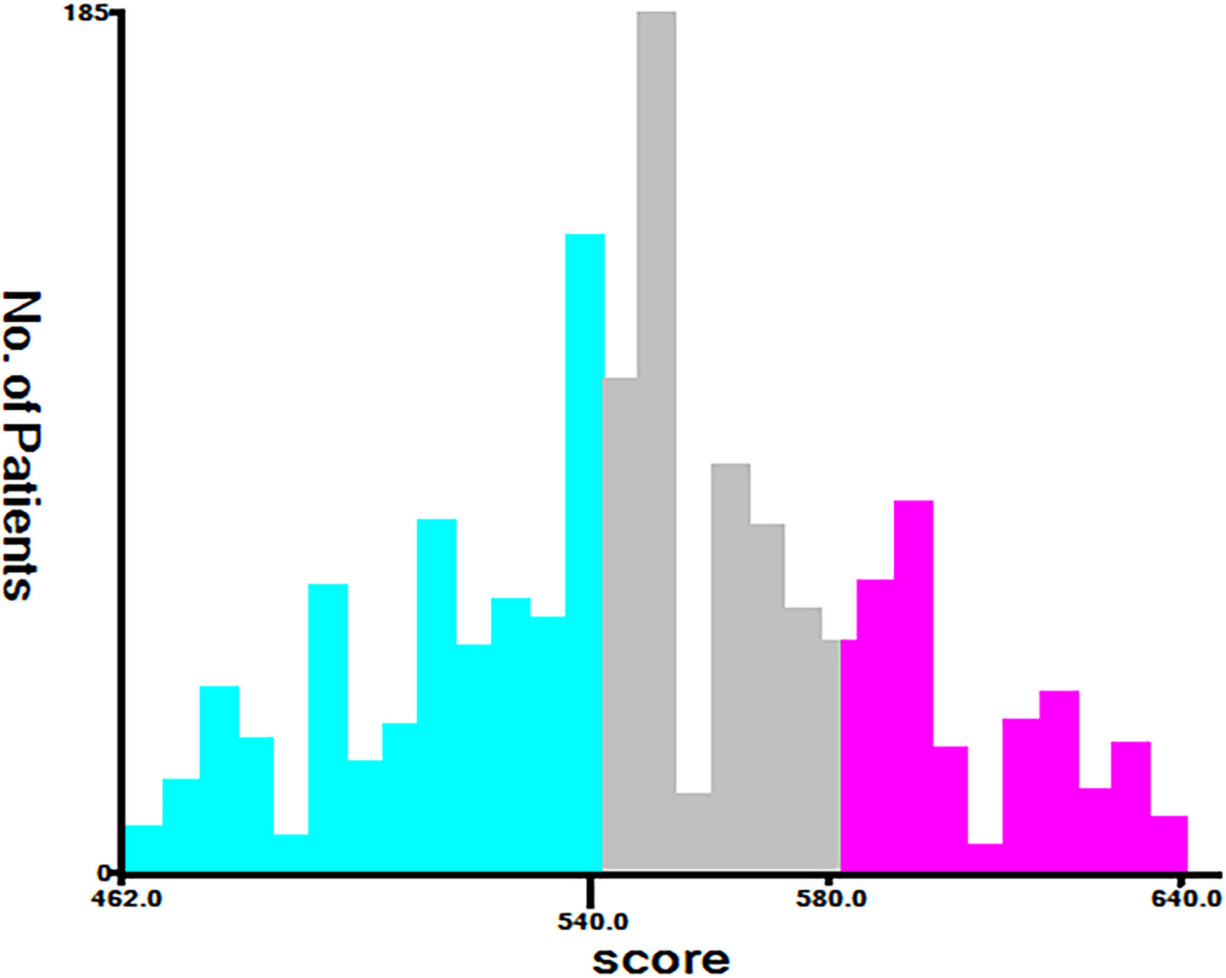
The basis for grouping new risk stratification (Cut-off point selected using X-tile).

**Figure 8:**
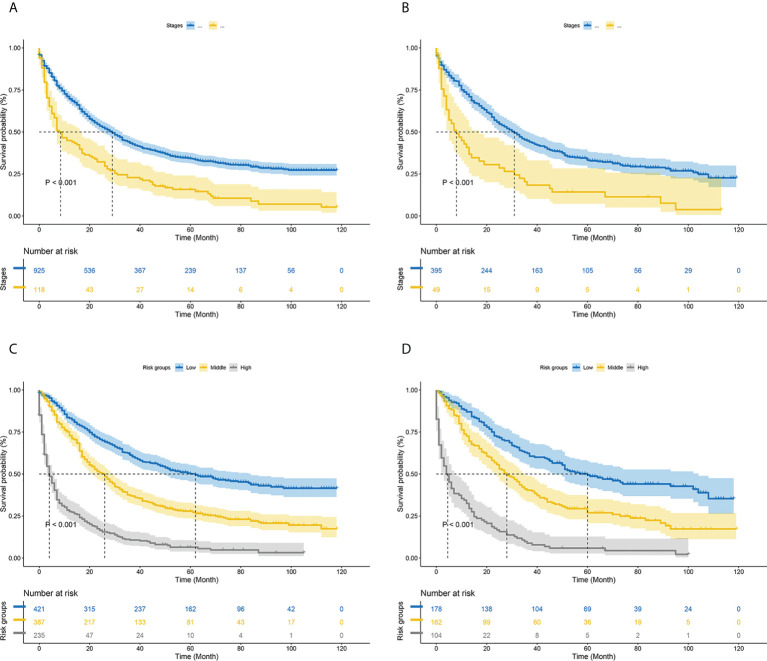
Kaplan–Meier curves of cancer-specific survival for new risk classification and the AJCC tumor staging **(A)** The AJCC tumor staging in the training cohort; **(B)** The AJCC tumor staging in the validation cohort; **(C)** The new risk classification in the training cohort; **(D)** The new risk classification in the validation cohort.

According to the analysis results of X-tile software, all patients were re-divided into three groups (low risk: total score <540, middle risk: 540≤ total score <580, and high risk: total score ≥580) based on the nomogram (**Figure 7**). Kaplan-Meier curves suggested that the new classification system has a more satisfactory capability than traditional AJCC staging (**Figure 8**).

## Discussion

HCC is one of the most common malignant tumors worldwide. MVI still exists in the residual liver after radical resection of liver cancer, and the detection rate is about 40% (26). HCC combined with MVI is more likely to invade blood vessels and lymphatic vessels, leading to early tumor spread and metastasis, which is an important factor predicting the prognosis of HCC (8, 9). Therefore, this study aimed to construct a nomogram to predict the prognosis of patients with HCC complicated with microvascular invasion. According to the results of multivariate COX regression analysis of the model group, 9 predictors including age, tumor size, pathological grade, AFP, radiation, chemotherapy, AJCC stages and surgery were included in the nomogram model that predicted the prognosis of MVI in HCC patients. The verification of the nomogram shows that the standard curve in the calibration curve of the model group and the validation group is basically close to the calibration prediction curve, indicating that the model has good discrimination and calibration ability. Based on the total score, patients were divided into low risk, middle risk, and high risk groups (the best cut-off values were selected by the X-tile software). In addition, Kaplan-Meier curves also showed that the new nomogram have more satisfactory discriminative power than traditional staging systems in stratifying the prognosis of HCC patients with MVI.

Previous studies have proposed some factors that may affect CSS in HCC patients complicated with MVI, such as age, tumor size, pathological grade and adjuvant therapy. However, these studies did not include additional clinical variables that affect the prognosis of HCC patients with MVI, and the small size of the total number of cases included in these studies inevitably biased the findings (27, 28). This study takes these factors into full consideration. Age and AFP are known significant risk factors, with lower AFP levels and longer median survival in older patients (29–31). Nathan (32) and Hirokawa (33) reported that tumor size is an independent risk factor for prognosis in HCC patients with MVI, which is consistent with the findings in this study that tumor diameter greater than 5 cm was an independent risk factor. The abnormal differentiation of tumor is one of the main characteristics of cancer cells, which is characterized by abnormal function and naive shape, and has strong proliferation and infiltration ability. Poorly differentiated cancer cells tend to have stronger vascular invasion capabilities, so the pathological grade is associated with the prognosis of MVI-HCC (34). Surgical resection is currently the treatment of choice for HCC patients combined with MVI who are not eligible for liver transplantation. As can be seen in the nomogram, there is a clear survival benefit from surgery. Hepatectomy is generally considered an important measure to improve patient prognosis, and hepatectomy still has a significant survival advantage for partial BCLC stage B HCC (35). In solitary small hepatocellular carcinoma with MVI, surgery rather than radiofrequency ablation should be used for initial treatment, and overall and disease-free survival with anatomic resection is significantly better than limited resection (36, 37). Adjuvant therapy is a controversial factor. Postoperative adjuvant TACE can reduce the tumor recurrence rate and help to improve the overall survival rate and tumor-free survival rate of liver cancer patients with MVI or multinodular tumors (38, 39). In patients with early (≤12 months) recurrence of mvi-positive HCC, TACE provided better overall survival than surgery or RFA (40). However, in many retrospective studies, postoperative adjuvant radiotherapy improved local control of HCC patients with MVI and was superior to TACE or conservative therapy, especially for patients with non-anatomical liver resection (NAR) (41, 42). In this study, our nomogram showed that adjuvant chemotherapy and radiotherapy could improve the postoperative prognosis of HCC patients with MVI.

The nomogram in this study integrates more factors into a quantitative model and has been shown to be superior to the AJCC in predicting prognosis and planning clinical strategies. Generally, the AJCC staging system has been closely related to OS. However, different outcomes were observed among patients in the same stage, which may be related to age, tumor size, adjuvant therapy and other factors. Therefore, we compared the nomogram involving multiple variables with tumor staging based on conventional AJCC criteria. The positive IDI and NRI of the nomogram demonstrated that the nomogram had superior predictive performance than the AJCC staging criteria alone. Furthermore, DCA revealed that the nomogram had better clinical utility and benefit in predicting CSS than conventional AJCC criteria. We established a risk stratification system, which could distinctly classify all patients into three risk prognostic groups according to their nomogram TPs. The cancer-specific survival curves presented that the new risk stratification system was superior to the conventional AJCC criteria in identifying different risk groups. Due to the poor prognosis of high-risk patients, we should pay more attention to patients with TP>580. These results suggest that the current nomograms are convenient and useful to assess the prognosis of HCC patients with MVI.

Despite the good performance of the nomogram, there are some limitations of this study. First, SEER did not publish data on virus-related variables, markers of inflammation, surgical margins. Therefore, these variables were not evaluated in this study. Second, the accuracy of the nomogram may be further enhanced by incorporating preoperative MVI prediction models and incorporating other prognostic biomarkers (43, 44). Finally, this study requires multicentre clinical data from other countries to verify the application value of the nomogram.

## Conclusions

Based on the seer database, a comprehensive nomogram and an innovative risk stratification system were established to predict CSS for HCC patients with MVI. Internal validation proved that the risk classification system possesses promising application capabilities. Compared to AJCC staging, the system is valid to differentiate between different risk groups, which may serve as an efficient tool for individualised treatment of patients. However, this result requires validation with data from other countries.

## Data availability statement

The original contributions presented in the study are included in the article/**Supplementary Material**. Further inquiries can be directed to the corresponding authors.

## Author contributions

Conceptualization: DY, MZ, XX, YS. Data curation: GZ, YH, XX. Formal analysis: JP, XX, YS. Writing-original draft: DY, MZ, FZ. Writing-review and editing: YD, YH. All authors contributed to the article and approved the submitted version.

## Conflict of interest

The authors declare that the research was conducted in the absence of any commercial or financial relationships that could be construed as a potential conflict of interest.

## Publisher’s note

All claims expressed in this article are solely those of the authors and do not necessarily represent those of their affiliated organizations, or those of the publisher, the editors and the reviewers. Any product that may be evaluated in this article, or claim that may be made by its manufacturer, is not guaranteed or endorsed by the publisher.
